# Malaria relevance and diagnosis in febrile Burkina Faso travellers: a prospective study

**DOI:** 10.1186/1475-2875-12-270

**Published:** 2013-08-02

**Authors:** Stéphanie Schrot-Sanyan, Sylvie Gaidot-Pagnier, Ahmed Abou-Bacar, Sodiomon Bienvenu Sirima, Ermanno Candolfi

**Affiliations:** 1 Institut de Parasitologie et de Pathologie Tropicale, EA 4438, Université de Strasbourg, Strasbourg, France; 2 Centre National de Recherche et de Formation sur le Paludisme, Ministère de la Santé, Ouagadougou, Burkina Faso

**Keywords:** Malaria, QBC, HRP2, Fever, Burkina Faso

## Abstract

**Background:**

There is a lack of information regarding the epidemiology of malaria among travellers from non-malaria endemic countries to Sahelian areas. The literature provides general statistics about imported malaria in industrialized countries or extensive recommendations about fever management, but none of these recommendations are applicable to developing countries.

**Methods:**

The aim of the study was to evaluate the aetiologies of fever, malaria prevalence, and best diagnostic methods in a population of 306 non-malaria endemic travellers who, over a one-year period, consulted the French embassy’s Centre Médico-Social in Ouagadougou (Burkina Faso) for fever. All patients underwent a clinical examination, a questionnaire, and three different malaria tests: thick blood film, QBC-test and HRP-2-based rapid diagnostic test.

**Results:**

Fever was caused by malaria in 69 cases (23%), while 37 (12%) were due to Pneumonia and 35 cases (8%) to ENT infections. Fever remained unexplained in 87 patients (51.3%). Malaria prevalence varied throughout the year: about 90% of malaria cases were diagnosed during and after the rainy season, between July and December, with up to 50% malaria prevalence for fever cases in October. Malaria diagnosis based solely on clinical signs, combined or not, leads to about 80% of unnecessary treatments.Although anti-malarial chemoprophylaxis was used in only 69% of short-stay patients (who travelled for less than three months), this was effective. Under local conditions, and using blood film examination as the reference method, the QBC test appeared to be more reliable than the HRP-2-based rapid diagnostic test, with respective sensitivities of 98.6% versus 84.1%, and specificities of 99.6% versus 98.3%.

**Conclusions:**

Reliable biological diagnosis of malaria among travellers from non-malaria endemic countries in Sahelian areas is necessary because of low malaria prevalence and the poor performance of clinical diagnosis. A fever during the first half of the year requires investigating another aetiology, particularly a respiratory one. Malaria chemoprophylaxis is efficient and must not be overlooked. The QBC test appears to be the most reliable diagnostic test in this context.

## Background

In Burkina Faso, a Sahelian country in West Africa, malaria transmission is holoendemic, with a very low permanent transmission and a very strong seasonal component [1-4]. Each year, there are approximately 1,5 million malaria cases and 40,000 victims; 90% of them are children under 15 years of age [5-7]. Approximately 20,000 French people travel to Burkina Faso each year, and 3,200 settle there permanently [8].

There are some general statistics regarding malaria importation in industrialized countries. With a general prevalence for malaria in sub-Saharan Africa travellers of one to four per 1,000 [9,10], the prevalence of fever due to malaria after travelling to a tropical country is quite variable, ranging from 27% to 52% [11-15], and malaria among patients consulting in Europe after a travel in sub-Saharan Africa is 20% in 2009 [16]. However, this data was collected in hospitals or specialist settings, or from a specific population. The different ”tropical” destinations, though varied, were not specifically identified in these studies. Burkina Faso often appeared in the “sub-Saharan Africa” category, which brought together sanitary situations as varied as those in Sahelian and equatorial areas. The literature provides different recommendations about fever management and malaria suspicion after travelling to a tropical country [17-20]. These recommendations are very extensive, but are not applicable to a developing country, where physicians are rare and tests not always available or reliable: if malaria diagnosis is unproblematic in Europe, where laboratories are required to provide a diagnosis within 2 hours, the same cannot be said for malaria diagnosis in endemic countries [21-31]. Then, the only safe solution for patients is to use an “Emergency Standby Treatment for Malaria” [32].

But a medical structure must be able to establish a reliable diagnosis and trust its own assays.

The aim of this study was to determine malaria prevalence and other main aetiologies of fever among febrile patients from non-malaria endemic countries, according to the length of their stay in Burkina Faso. Microscopic quantitative buffy coat test (QBC), which was used during consultations, was compared to the non-microscopic rapid diagnostic test (RDT), which targets the histidine-rich protein-2 (HRP2) of *Plasmodium falciparum*.

## Methods

### Inclusion criteria

This study was conducted at the French embassy’s Centre Médico-Social (CMS) in Ouagadougou (Burkina Faso). This is a small primary health centre that treats predominately French embassy staff and travellers living in or passing through Burkina Faso and coming from non-malaria endemic countries. From this population, patients over 15 years of age who had spent the first five years of their life outside of a malaria-endemic area and had “suspicion of malaria” between July 2006 and July 2007 were recruited. “Suspicion of malaria” was defined as “febrile syndrome” in the last 48 hours, for fever remained the most sensible clinical sign of malaria [33,34]. “Febrile syndrome” was defined as an uncorrected axillary temperature over 37.5 °C, as measured at the air-conditioned CMS, at home, or by acute febrile signs including shivers, hot flashes, and sweating.

### Data collection

During the consultation, an oral consent was informed for each patient. A questionnaire was completed with the patient with the following information:

Malaria chemoprophylaxis;

Anti-malarial and antipyretic treatment taken over the last few days;

Travels outside Burkina Faso over the last few weeks.

All patients underwent an examination. Uncorrected axillary temperature of each patient prior to the clinical examination was measured. Patients with temperature above 38.0°C were considered as “febrile on examination”.

Using a sterile lancet, blood samples were collected via finger prick for three malaria tests:

QBC-test: 50-65 μl of blood was drawn into an acridine orange-coated heparinized capillary tube, handled according to the manufacturers’ recommendations [35], and read on site,

HRP2-based RDT (Palutop®, from the Alldiag, Strasbourg, France): one drop of blood with solvent was read on site under oil immersion after 15 minutes according to the manufacturers’ recommendations [36],

Thick and thin blood films were used to establish a reference diagnosis: one slide with four blood drops [three for the Giemsa-stained thick film (GTF) and one for the thin blood film]. These slides were coloured once a week with Giemsa at the Centre National de Recherche et de Formation sur le Paludisme in Ouagadougou, a public health research centre in Ouagadougou. The slides were read at the end of the study following the Centre’s protocol: the slides were examined by two experienced researchers (and possibly by a third expert in case of disagreement between the two. Parasitaemia was calculated on the thick film by the number of trophozoites for 10 leucocytes, and the species was determined on the thin film when the thick film was positive. About 10% of these slides (taken at random) and all of the conflict cases (in which one of the three tests showed a different result from the other two) were re-read (complete reading of the thick and thin films) at the Institut de Parasitologie et de Pathologie Tropicale de Strasbourg in order to establish a final reference diagnosis.

### Diagnosis and patient management

This study was non interventional. Since the final reference diagnosis was not known at the time of consultation, the QBC-test result was taken into account so as to determine whether to treat the patients for malaria, as the physicians of the CMS usually did. When the QBC-test was positive, patients were treated according to national guidelines:

Non-complicated malaria: association of lumefantrine (120 mg) and artemether (20 mg), 6 times four tablets every 12 hours;

Complicated malaria [37]: 8 mg/kg of intravenous quinine every 8 hours, with an initial bolus of 16 mg/kg and relay with oral quinine 48 hours after the last febrile seizure for 10 days of treatment, at the same dosage.

When the QBC-test was negative, further investigations were eventually carried out by the physicians of the CMS, according to the clinical status, to determine the aetiology of the observed febrile syndrome, such as urinary cultures for pyelonephritis and chest x-rays for pneumonia. No anti-malarial treatment was initiated without a positive QBC test.

### Data analysis

For statistical analysis, Chi-squares (χ^2^) or Student-t-tests (t-test) were used, as appropriate. Bilateral analysis was computed, and the significance level was set at 0.05.

## Results

Over a one-year period, informed consent was obtained from 306 patients consulting for febrile syndrome: 149 males and 157 females. Patients were separated into two groups according to the amount of time spent in Burkina Faso: patients that stayed longer than three months were labelled “resident” (202) and those that stayed less than three months were labelled “traveller” (104).

The distribution varied throughout the year. “Travellers” were most often present (and, therefore, attended medical consultation) during summer and Christmas vacations or during the international film festival in February (FESPACO), while “residents” were present year-round. Two peaks of consultation for febrile syndrome were noted in the “resident” population: the first in October, just after the rainy season, and the second one in February, before the start of the hot season.

### Fever on examination

We first evaluated antipyretics as potentially confounding factor, following the Mantel-Haenszel method. The stratificated ORs of patients with and without antipyretics were compared to determine if an effect modification or interaction was indeed taking place between those two variables. There was no statistical interaction neither with patients considered “febrile on examination” (OR O,91, [0,47-1,74]) nor with malaria (OR 0,76 [0,36-1,62]).

Of the 57 patients who were “febrile on examination” (patients with an uncorrected axillary temperature over 38.0°C), only 22 had malaria (38.6%), which is a significantly higher percentage than that of the 47 malaria cases (18.9%) among the 249 “non-febrile patients on examination” (χ^2^, *p < 0.001*).

### Malaria and other aetiologies of fever throughout the year

Aetiologies of fever are reported in Table 1. Of the 306 patients, 69 were diagnosed with malaria (22.6%): 67 *P. falciparum* (97.1%) including two mixed infections with *Plasmodium malariae*, and one with *P. malariae* only. In the last case, no species could be determined, due to an extremely low parasitaemia (post-treatment diagnosis, with very low positive GTF and negative thin blood film).

**Table 1:** Fever aetiologies in 306 adults from non-malaria endemic countries living or travelling in Burkina Faso

Undefined fever	
With acute digestive symptomatology	87 (28.4%)
Without acute digestive symptomatology	70 (22.9%)
Malaria	69 (22.6%)
Pneumonia	37 (12.1%)
ENT infections	35 (7.8%)
Pyelonephritis	4 (1.3%)
Erysipelas	4 (1.3%)
Total	306 (100%)

**Figure 1:**
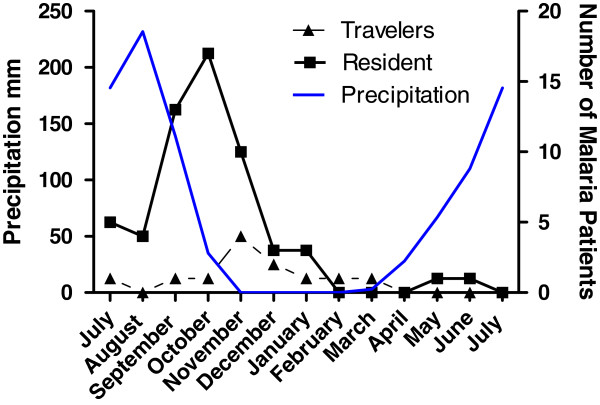
**Number of malaria cases in adults from non**-**Malaria endemic countries living or travelling in Burkina Faso who presented with acute fever during the next year.**

**Figure 2:**
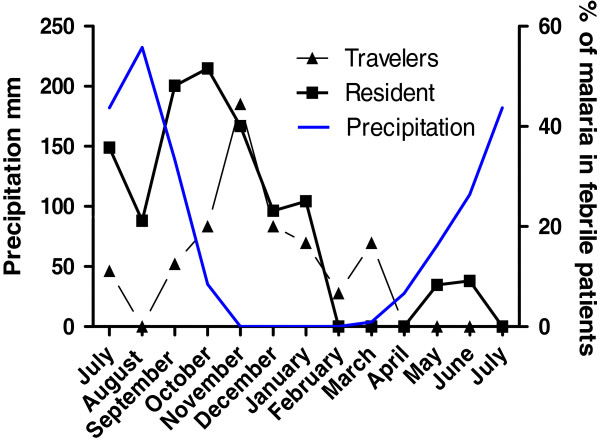
**Malaria prevalence in adults from non**-**Malaria endemic countries living or travelling in Burkina Faso and presenting with acute fever.**

Most malaria cases (88.4% of diagnoses [Figure 1]) were concentrated between July and December, with a peak in October-November, approximately one month after the precipitous peak of the rainy season. The maximal malaria prevalence (nearly 50%) was in October-November among both “travellers” and “residents” (Figure 2).

In January, three patients diagnosed with malaria reported having travelled outside Burkina Faso in the previous month (two to Ghana and one to Mali near the river Niger), as well as one in February (Mali).

### Malaria and chemoprophylaxis

Following the national and international recommendations, a long-term prophylaxis was not recommended to the residents, but only 69% of the travellers correctly took one. Only one of them (a “traveller”) who took chemoprophylaxis correctly suffered from malaria (Table 2). This young, 20-year-old patient took a prophylaxis based on a chloroquine and proguanil association in accordance with current recommendations [38] (level 2 in 2006) of the Institut de Veille Sanitaire, the French institute of public health. She was travelling in the southern part of Burkina Faso that borders the Ivory Coast. A higher anti-malarial drug resistance was recorded in Ivory Coast and a different prophylaxis is recommended (level 3, mefloquine or a proguanil and atovaquone association).

**Table 2:** Malaria prevalence according to the length of stay and malaria chemoprophylaxis in 306 febrile adults from non-Malaria endemic countries living or travelling in Burkina Faso

		**Non malaria**	**Malaria**
Journeys under 3 months	With adapted and well-taken chemoprophylaxis	71	1
	*Malaria prevalence*		*1.4%*
	Without appropriate chemoprophylaxis	21	11
	*Malaria prevalence*		*34.4%*
Journeys over 3 months	With adapted and well-taken chemoprophylaxis	27	0
	*Malaria prevalence*		*0%*
	Without appropriate chemoprophylaxis	118	57
	*Malaria prevalence*		*32.6%*

### Clinical signs and their association with malaria

Five categories for patient symptoms were determinate: digestive (vomiting, abdominal pain, diarrhoea, etc.), ear-nose-throat (ENT) (rhinitis, pharyngitis, odynophagia…), respiratory, urinary, and cutaneous. Those that did not correspond with any of these categories were considered “isolated fevers”. Table 3 shows the signs’ prevalence by malaria cases and non-malaria cases.

**Table 3:** Symptoms associated with fevers in 306 adults travelling in Burkina Faso, regardless of the link to malaria

**Symptom**	**Non-malaria patients**	**Malaria patients**	**Total**
	**237**	**69**	**306**
Isolated fever	68	54	122
*Prevalence*	*28.7%*	*78.3%*	*39.9%*
Digestive symptoms	88	9	97
*Prevalence*	*37.1%*	*13.0%*	*31.7%*
ENT symptoms	37	5	42
*Prevalence*	*15.2%*	*7.3%*	*13.7%*
Respiratory symptoms	36	1	37
*Prevalence*	*15.2%*	*1.5%*	*12.1%*
Urinary symptoms	4	0	4
*Prevalence*	*1.7%*		*1.3%*
Cutaneous symptoms	4	0	4
*Prevalence*	*1.7%*		*1.3%*

The performance of the best symptom or association of symptoms for malaria diagnosis in this study is displayed on Table 4. Isolated fever was the most frequent clinical sign for malaria (78.3%), but more than half of these isolated fevers were related to another origin. There were significantly more fevers on clinical examination in the malaria group.

**Table 4:** Statistical value of clinical signs, association of clinical signs and malaria tests (QBC and HRP2-based RDT) for the diagnosis of malaria, according to the results of the thick and thin Giemsa-stained blood films, considered to be the gold standard method (with 95% confidence intervals for sensibility and specificity)

	**Sensibility**	**Specificity**	**Positive predictive value**	**Negative predictive value**
Isolated fever	78.3%	71.3%	44.3%	91.9%
	[68.5 – 88.0]	[65.6 – 77.1]		
Fever present on examination	31.9%	85.2%	38.6%	81.1%
	[20.9 – 42.9]	[80.7 – 89.8]		
Isolated fever present on examination	26.1%	96.2%	66.7%	81.7%
	[15.7 – 36.5]	[93.8 – 98.6]		
Digestive symptoms	13.0%	62.9%	9.3%	71.3%
	[5.1 – 21.0]	[57.2 – 69.4]		
QBC	98.6%	99.6%	98.6%	99.6%
	[9.7 – 100]	[98.8 – 100]		
HRP2	84.0%	98.0%	92.0%	95.0%
(for *P. falciparum*)	[75.4 – 92.7]	[96.7 – 99.9]		

### Malaria diagnosis

To predict the performance of on-the-spot, available tests, namely the HRP2- based RDT and QBC-tests, these tests were performed in addition to thick and thin blood film examinations for each patient. The results of these three tests were coincident in 94% of the patients (289/306). The results of the tests’ diagnosis performance are summarized in Table 4.

## Discussion

### Malaria diagnosis

The QBC-test is quick and easy to use: every physician was trained for approximately one hour, and the test’s complete implementation took less than 10 minutes. The main inconvenience was the material: the initial investment is important (centrifuge, microscope with oil immersion). In a clinical laboratory in Ouagadougou in 2013, the cost of a QBC-test for the patient is between 1,500 and 2,000 XOF (2–3 €); this not much more expensive than for a GTF who will cost 1,000 to 1,500 XOF (1,5 to 2 €) [39]. The logistics (management of colouring agents and colourings) are less binding comparing to a GTF, because every test can be analysed individually. The Becton-Dickinson company, which developed this technique, stopped marketing the test’s in 2006 [40], but the assay commercialization was taken over by QBC-Diagnostics, which continues to develop new and less cumbersome devices [41]. The capillary tube enables a blood cell count obtained in a few minutes, which may be especially helpful when the fevers are unrelated to malaria, as observed in approximately 80% of cases.

The QBC-test offers more than a reliable performance: sensitivity and positive predictive value over 98%, and specificity and negative positive value over 99%. These figures are much superior to those found in the local GTF-using laboratories [27-29]: there was only one false-positive and one false-negative, both observed during post-treatment. However, the test did not allow identifying the species in the only case of *P. malariae* infection. This likely caused overtreatment via artemisinin-derivatives when chloroquine treatment would have been sufficient. Nonetheless, the benefit/risk balance remains much more favourable for the QBC-test, when more than 97% of the malaria cases were due to *P. falciparum*.

RDT was significantly overall less sensitive than the QBC, as confirmed by several studies [42,43]: the number of false-negatives by antigen HRP2 detection is high (15.9%). RDT remains useful only under conditions in which it is the only test available, and the results must be interpreted carefully: a negative test must be repeated in the absence of clinical improvement. A RDT costs between 4,000 to 10,000 XOF (6 to 15 €) in Ouagadougou in 2013.

The RDT remains an asset in retrospective diagnosis, when PCR is not available: 10% of our patients, prior to fever consultation, had already undergone malaria treatment. In one of these cases, QBC did not diagnose malaria, whereas the RDT would have been able to make the diagnosis. Four other patients, who had also already undergone treatment, exhibited negative direct microscopic test (QBC and GTF), along with a positive RDT; we wonder whether these were authentic malaria cases, though masked by treatment. This would have changed patient management: in the case of proven malaria, post-treatment observation would be increased, and in the case of a negative test, we would direct our search towards another aetiology.

### Malaria prevalence and other aetiologies of fever

Malaria was the second highest fever diagnosis behind unexplained fevers, with a prevalence from 22.6%. The rate among African residents in Ouagadougou was not different, and was between 17% and 20% [44]. The malaria prevalence among “residents” with febrile syndrome was 28.2% on average, which was significantly higher than that among “travellers” (11.5%, χ^2^, *p < 0.001*). With respect to the malaria cases, 82.6% were diagnosed among “residents”, even though they represented only 66% of total patients.

Pneumonias were the third aetiology of fever (13.8%). They were especially prevalent at the beginning of the hot season, when the Harmattan, a very dry, hot, and sandy wind blows from the desert. Thus, the Harmattan may account for the second peak in February fever distribution. This phenomena, which is well documented in Burkina Faso [44], also facilitates meningococcal diffusion. For Africans living in Ouagadougou, pneumonias are the first aetiology of fever and represent 27.6% of all fevers [44].

### Chemoprophylaxis

Only one malaria case was recorded in a “traveller” under appropriate and successfully administered chemoprophylaxis, according to the current French recommendations in 2006. These recommendations have since evolved, which is in accordance with this result, but only 69% of the travellers correctly took one.

### Clinical signs associated to the malaria

With a malaria prevalence of only 22.6%, a systematic treatment by febrile syndrome such as “Emergency Standby Treatment”, as recommended for travellers going to remote places where access to medical care is unlikely to be within 24 hours, will lead in Burkina Faso to 77.4% of unnecessary treatments. A clinical diagnosis based on isolated fever, with a negative predictive value (NPV) of 91.9% and a positive predictive value (PPV) of 44% (Table 4), would result in 56% of unnecessary treatments and 22% of undiagnosed malaria infections. Such a clinical diagnosis would cause a diagnosis delay with potentially serious consequences. Other important clinical signs or combinations of signs do not allow us to justify a presumptive treatment. Isolated fever, present at the time of the examination, would be a strong factor of “suspicion of malaria” with a PPV of 66.7%.

A reliable and systematic test for malaria diagnosis is necessary to judiciously dispense malaria therapies to a population of non-immune adults staying in an endemic area. The low malaria prevalence among fevers, as well as the clinical examinations’ lack of sensibility and specificity, do not justify the risk of severe and unwanted side-effects, treatment resistance, heavy financial burdens associated with using new molecules, such as artemisinin-derivatives [24,45,46], as well as delays in the diagnosis of other serious diseases [47-50].

**Figure 3:**
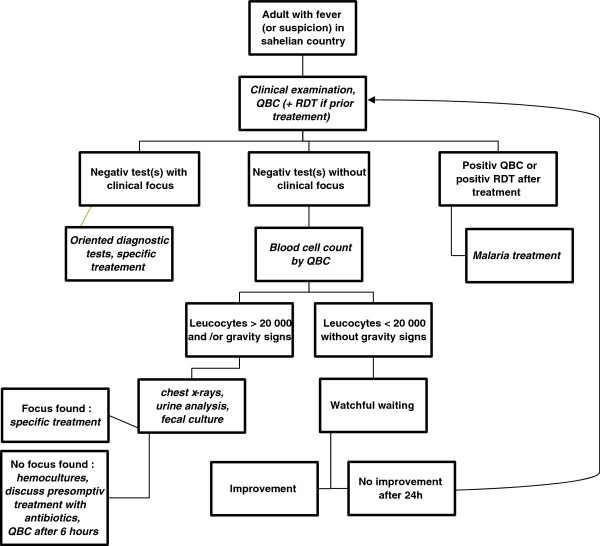
Management recommendation for febrile adults from non-malaria endemic countries living or travelling in Sahelian countries.

Based on this data, Figure 3 attempted to define a decision algorithm in order to help physicians working in Burkina Faso*.*

## Conclusions

The two most frequent fever pathologies found in adults travelling to Ouagadougou, namely digestive tract infections and malaria, should be the object of information and preventative measures prior to departure. For malaria, chemoprophylaxis proved to be effective in this study. However, only 69% of short-stay patients effectively took chemoprophylaxis. Travel to high-risk areas is increasing, and it is becoming increasingly important for physicians to provide good advice prior to departure.

At the facility, all patients with fever or “suspicion of fever” must systematically be evaluated with a reliable malaria test prior to treatment. In this Sahelian context, the QBC seems to be the most reliable diagnosis test; the RDT still remains a feasible testing option, especially in the case of prior treatment. However, since malaria has an especially low prevalence in dry season, other aetiologies, such as pneumonias, must be considered without delay.

## Abbreviations

χ2: Chi-squared test; ENT: ear, nose, and throat; GTF: Giemsa-stained thick film; NPV: Negative predictive value; OR: odds ratio; PPV: Positive predictive value; QBC: Quantitative Buffy Coat; RDT: Rapid Diagnostic Test; t-test: Student’s test.

## Competing interests

The authors have no conflict of interest to declare.

## Authors’ contributions

SSS and SGP with help of EC participated in the study design and collected the samples; SSS conducted the review and analysis and wrote the first draft of the manuscript. AAB and SBS performed the GTF examination. All authors took part in the preparation and final approval of the manuscript.
